# Computational fluid dynamics calculations in inferior turbinate surgery: a cohort study

**DOI:** 10.1007/s00405-023-08058-x

**Published:** 2023-06-21

**Authors:** Jaakko Ormiskangas, Olli Valtonen, Teemu Harju, Markus Rautiainen, Ilkka Kivekäs

**Affiliations:** 1 Faculty of Medicine and Health Technology, Tampere University, Tampere, Finland; 2 Faculty of Engineering and Natural Sciences, Automation Technology and Mechanical Engineering Unit, Tampere University, Tampere, Finland; 3 Department of Otorhinolaryngology - Head and Neck Surgery, Tampere University Hospital, Tampere, Finland

**Keywords:** CBCT, CFD, Nasal cavity, Inferior turbinate, Mucous membrane

## Abstract

**Purpose:**

To investigate how the results of nasal computational fluid dynamics (CFD) simulations change due to inferior turbinate surgery and how the results correlate with patient specific subjective assessment and volumetric results in the nasal cavities.

**Methods:**

The steady inspiratory airflow of 25 patients was studied pre- and postoperatively with heat transfer from the mucous membrane by performing CFD calculations to patient-specific nasal cone beam computed tomography images. These results were then compared to the severity of the patients’ nasal obstruction Visual Analogue Scale (VAS) and Glasgow Health Status Inventory assessments, and acoustic rhinometry measurements.

**Results:**

Total wall shear forces decreased statistically significantly (*p* < 0.01) in the operated parts of the inferior turbinates. Patients’ subjective nasal obstruction VAS assessment changes between the pre- and postoperative conditions correlated statistically significantly (*p* = 0.04) with the wall shear force results.

**Conclusion:**

Inferior turbinate surgery lead to decreased total wall shear force values postoperatively. Changes in subjective nasal obstruction VAS results against total wall shear force changes between the pre- and postoperative conditions were statistically significant. CFD data have a potential to be used for the evaluation of nasal airflow.

## Introduction

Currently, clinicians have only rudimentary objective tools at their disposal for assessing the nasal cavities and nasal congestion. Unfortunately, the subjective assessment of nasal patency and quality of life provided by patients does not always correlate with current objective methods [1, 2]. At present, the most detailed objective information is obtained from computed tomography or cone beam computed tomography (CBCT). CBCT imaging provides full three-dimensional (3D) information on the nasal cavities, which is not obtained in rhinomanometric studies. In addition to the clinical objective methods currently used, a further detailed 3D analysis of the nasal cavities and corresponding nasal airflow can be obtained using Computational Fluid Dynamics (CFD) calculations. These calculations are based on flow governed by Navier–Stokes equations, which are solved using numerical methods. CFD calculations can also be used to obtain information that is useful in assessing the subjective nasal patency of patients. Literature reviews of these methods has recently been conducted [3, 4].

Previously, nasal airflow CFD studies have been conducted using various approaches. CFD calculations are subject to boundary conditions and modelling assumptions which can affect the results. The important boundary conditions include mucous membrane properties and surface temperatures, turbulence modelling and nasal airflow volume flow rates. Most previous studies have investigated inspiratory nasal airflow with constant patient specific- volume flow rates either pre- and postoperatively or preoperatively against healthy controls. The subjective responses of patients are usually analysed against calculated CFD results, such as heat transfer and wall shear stresses (WSS), which are then reported as meaningful objective CFD measures. Most studies of nasal airflow have used heterogenous patient cohorts in which septoplasty is often performed [5–9]. In these studies, the best correlations have been found between visual analogue scale (VAS) assessment from the most obstructed side of the nasal cavities and CFD results from the same side. Similar correlations have also been observed with NOSE surveys and VAS assessment against CFD results.

In the present study, we aim to investigate how CFD calculations can model patient specific VAS and quality of life (QOL) values as a function of flow variables in inferior turbinate surgery. In addition, acoustic rhinometry and volumetric measurements are investigated and compared to CFD results.

## Materials and methods

### Surgical procedure and follow-up visits

In the present study, 25 patients with chronic nasal obstruction caused by enlarged inferior turbinates were included. All included patients underwent radiofrequency thermal ablation (RFTA) treatment (Sutter RF generator BM-780 II, Freiburg, Germany) to the anterior parts of the inferior turbinates on both sides. The exclusion criteria of the study were chronic sinusitis, nasal polyps or other nasal pathologies. The surgical process is described in detail elsewhere [10]. The volumetric changes in the nasal cavities between the pre- and postoperative conditions of the surgical process have been previously reported by Valtonen et al. [11]. In the present study, patients were evaluated prior to surgery and at one year after surgery. During both visits, the patients were scanned with cone beam computed tomography (CBCT) (Planmeca Max, Planmeca, Helsinki, Finland). The following imaging parameters were used: 0.2 mm CT slice thickness, voxel size 0.2 mm, 90 kVp, 8 mA and 4 s radiation time. Additionally, patients were asked to fill out the Visual Analogue Scale (VAS) questionnaire both pre- and postoperatively to simultaneously evaluate the severity of the nasal obstruction on both sides of the nasal cavities. The VAS questionnaire assessed patient experiences from the previous 7 days (on a scale of 0–10, with 0 meaning completely open and 10 completely blocked). Furthermore, to assess the QOL, patients were also asked to fill in the Glasgow Health Status Inventory (GHSI) questionnaire from the previous 7 days. The GHSI questionnaire contains 18 questions measured with a 5-point Likert scale. These scores are then transformed to a scale ranging from 0 to 100. Higher scores in the GHSI indicate an enhancement in patient well-being and QOL. Additionally, acoustic rhinometry V2-5 cm (Acoustic rhinometer A1, GM instruments Ltd, Kilwinning, UK) results were also investigated pre- and postoperatively without vasoconstriction. However, one patient had missing acoustic rhinometry results. Therefore, acoustic rhinometry results were analysed with 24 patients. Institutional Review Board approval for the study (R13144) was obtained from Tampere University Hospital Ethics Committee, Tampere, Finland.

### CBCT data and CFD calculations

The aim of the present study was to study the VAS and GHSI assessments of patients as a function of the flow variables obtained from CFD calculations between the pre- and postoperative conditions in inferior turbinate surgery. The CBCT data of the chosen patients were saved to a file in Digital Imaging and Communications in Medicine (DICOM) format and downloaded to OnDemand3D™ software (version 1.0, CyberMed, Inc., Yuseong-gu, Daejeon, South Korea). The software was then used to create a 3D model of the air space in the nasal cavities from the nostrils to the nasopharynx. For 3D modelling, Hounsfield unit (HU) values from − 1000 to − 430 were used to represent the air space according to previous studies [2, 11–14]. The maxillary, frontal and sphenoidal sinuses were excluded from the study. Additionally, any small 3D modelling artefacts included in the 3D models by the software, were manually excluded from the structures of interest. The prepared 3D models were then saved in Standard Tessellation Language (STL) format. When necessary, any small artefacts still found in the 3D models were removed from the STL files using the open-source modelling software Blender v2.82a (Blender Foundation). Isolated air regions with no possible airflow were also removed and were not included in the CFD calculations or volumetric measurements.

The STL files were then meshed with open source OpenFOAM 5.0 software’s utilities blockMesh and snappyHexMesh. The meshing of wall boundary layers was made using absolute size refinements, whereas hexahedrical elements were used for the inner geometry with similar cell sizes in all regions. Mesh sizes were between four and six million cells in all calculations.

The same OpenFOAM 5.0 software was used for CFD calculations with a laminar and incompressible flow assumption. In previous studies, laminar calculations have been found to be suitable for nasal airflow. Uniform total pressure condition was applied in the nostrils. To study the airflow in the nasal cavities, we used a constant inspiratory flow rate that was also patient specific by determining the flow rate as a function of gender and weight [15]

1 $$\begin{gathered} {\text{For males:}}\,\dot{V} = \left( {1.36 \pm 0.10} \right)M^{{\left( {0.44 \pm 0.02} \right)}} \hfill \\ {\text{For Female:}}\,\dot{V} = \left( {1.{89} \pm 0.{40}} \right)M^{{\left( {0.{32} \pm 0.{06}} \right)}} \hfill \\ \end{gathered}$$

where $\dot{V}$ is flow rate (litres per minute) and $M$ is weight in kilograms. The steady-state inspiratory flow rates used in the calculations were twice the mean flow rate for each patient defined from Eq. (1). In addition, equal flow rates were used pre- and postoperatively for each patient. All calculations were conducted with heat transfer from the rigid walls with no-slip condition. Ambient air had a temperature of 20 °C. For our heat transfer calculations, the mucous membrane surface temperatures ${T}_{\text{mucous}}$ were adjusted to have a mean temperature of approximately 32 °C according to the following Equation:

2 $$q = h_{m} \left( {T_{{{\text{body}}}} - T_{{{\text{mucous}}}} } \right)$$

where $q$ is a local heat flux (W/m^2^) from the mucous membrane surface to airflow; ${T}_{\text{body}}$ is body temperature of 310.15 Kelvin (K); ${T}_{\text{mucous}}$ is a local mucous membrane surface temperature and ${h}_{m}$ is a uniform heat transfer coefficient across the mucous membrane in all positions of the nasal cavities. Coefficient ${h}_{m}$ was experimentally adjusted to ${50}\mathrm{W}/\left({\mathrm{m}}^{2}\mathrm{K}\right)$ to have similar mucous membrane surface temperatures as in the clinical measurements by Lindemann et al. [16]. Across the whole nasal cavity, Eq. (2) obtains approximately a mean mucous membrane surface temperature of 32 °C. However, when no local heat transfer is present, Eq. (2) obtains a mucous membrane temperature of 37 °C. To complete the calculation of a single 3D model, one week was required with 24 processors. The heat transfer calculation procedure presented here is similar to the study by Ormiskangas et al. [14].

### Data analysis

**Fig. 1:**
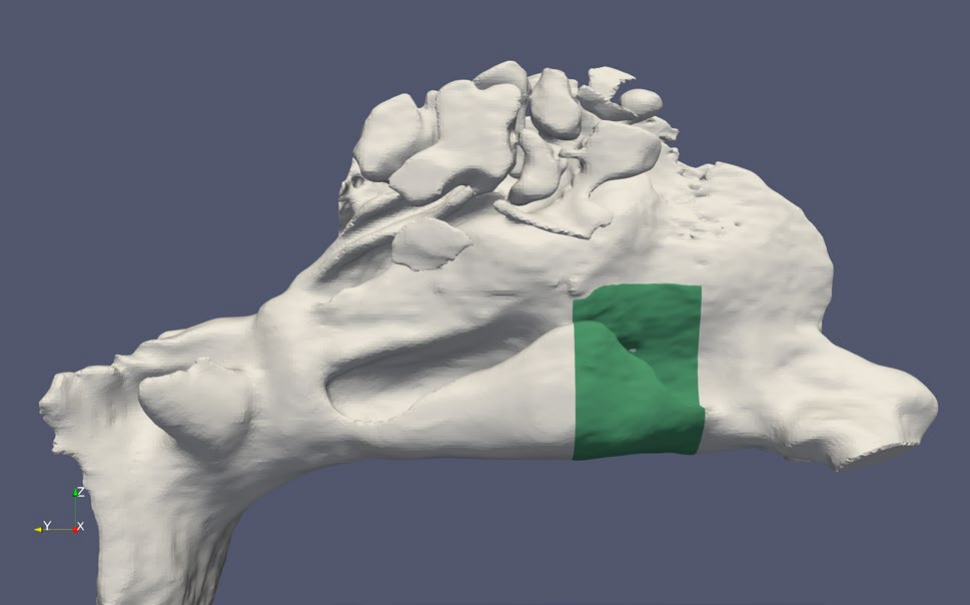
The preoperative nasal cavities for one patient. The operated anterior 5–20 mm parts of the inferior turbinates are marked in green

In a previous study, most volumetric changes from inferior turbinate surgeries have been found to occur in the anterior parts comprising 5–20 mm of the inferior turbinates [11]. Therefore, total heat transfer and magnitudes of wall shear forces were calculated at the surface of the mucous membrane in the operated anterior parts of the inferior turbinates **(**Fig. 1**)**. The anterior part comprised 5–20 mm area posterior from the anterior peak of the inferior turbinate. The uppermost limit of the inferior turbinates was set at the lowest level of the middle turbinates. All values were then integrated separately into total values in the operated anterior 5–20 mm parts of the inferior turbinates.

Pressure losses along the nasal cavities were calculated from the nostrils to the nasopharynx to assess the total effects of the inferior turbinate surgeries. Nasopharynx results were measured vertically at the bottom level of inferior turbinates airways since not all patients had much downstream geometry from the nasopharynx. In all our calculated cases, our mesh geometry was extended 50 mm downstream from the nasopharynx for computational reasons. The analysis was made to both sides of the nasal cavities simultaneously, as was the treatment and patient VAS assessment and GHSI questionnaire. The Wilcoxon signed-rank test was used to analyse the statistical significance of the results between the pre- and postoperative conditions. Spearman’s rank correlation was used to analyse univariate correlations between all results. The numerical post-processing of the acquired CFD data was performed with the open-source visualization and data analysis software ParaView 5.0.1.

## Results

**Fig. 2:**
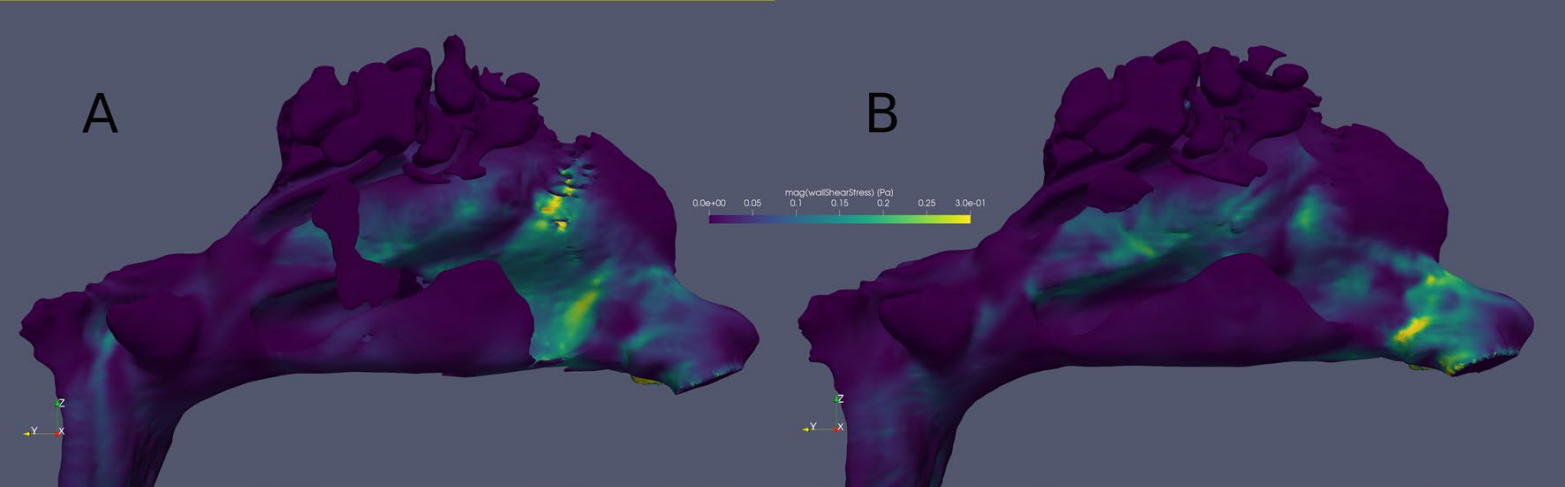
The pre- (**A**) and postoperative (**B**) wall shear stress (Pa) results for one patient

In the anterior 5–20 mm of the inferior turbinates, statistically significant decreases were found in total wall shear forces (*p* < 0.01) between the pre- and postoperative conditions (*n* = 25). However, no significant changes were found in the heat transfer results in the anterior parts of the inferior turbinates or in any of the results from the rest of the nasal cavity. (Table 1) The pre- and postoperative wall shear stresses are visually presented for one patient **(**Fig. 2**)**.

**Table 1:** Pre- and 12-month postoperative Computational Fluid Dynamics results and their changes

Calculated CFD results and changes				
	Wall shear forces in the anterior 5–20 mm parts of the inferior turbinates [N]	Heat transfer in anterior 5–20 mm parts of the inferior turbinates [W]	Wall shear forces outside of the anterior 5–20 mm parts of the inferior turbinates [N]	Heat transfer outside of the anterior 5–20 mm parts of the inferior turbinates [W]
Preoperative median (IQ25–IQ75)	3.22*10^–4^ (2.53*10^–4^ to 4.25*10^–4^)	0.51 (0.40 to 0.63)	1.93*10^–3^ (1.35*10^–3^ to 2.17*10^–3^)	3.22 (2.82 to 3.61)
Postoperative median (IQ25–IQ75)	1.62*10^–4^ (1.36*10^–4^ to 2.27*10^–4^)	0.46 (0.40 to 0.49)	1.82*10^–3^ (1.19*10^–3^ to 2.04*10^–3^)	3.01 (2.63 to 3.63)
Change median (IQ25–IQ75)	− 1.12*10^–4^ (− 2.54*10^–4^ to − 6.03*10^–6^)	− 0.05 (− 0.13 to 0.03)	− 1.19*10^–4^ (− 7.27*10^–4^ to 1.31*10^–4^)	− 0.06 (-0.28 to 0.06)
Change *p*-value	< 0.01	0.09	0.14	0.14

Both sides of the nasal cavities are analysed together. The Wilcoxon signed-rank test was used to calculate two-sided *p*-values

In the anterior parts of the inferior turbinates, a statistically significant negative correlation was found between total wall shear force values (*p* < 0.001) and volumetrically measured airway volumes (*n* = 50) when all values were pooled (pre- and postoperative, *n* = 50). In the same area, no statistically significant correlation was found between heat transfer and volumetrically measured airway volumes. Furthermore, there were no statistically significant correlations between total wall shear forces and heat transfer in the operated parts of the inferior turbinates against acoustic rhinometry V2–5 cm results. For patients’ subjective assessment no statistically significant correlations were found between total wall shear force or total heat transfer in the operated parts against nasal obstruction VAS or GHSI assessments (Table 2).

**Table 2:** Univariate Spearman correlations between calculated CFD values (total wall shear forces and total heat transfer) from the anterior 5–20 mm parts of the inferior turbinates and the subjective and volumetric measures

Spearman correlations between CFD values and other subjective and objective measures in the anterior 5–20 mm parts of the inferior turbinates								
	Wall shear forces against VAS	Heat transfer against VAS	Wall shear forces against GHSI	Heat transfer against GHSI	Wall shear forces against V2–5 cm	Heat transfer against V2–5 cm	Wall shear forces against air volume	Heat transfer against air volume
Spearman’s rho	0.19	0.17	− 0.08	− 0.01	− 0.26	0.12	− 0.68	− 0.24
*p*-value	0.18	0.24	0.56	0.94	0.07	0.41	< 0.001	0.09

Both sides of the nasal cavities are analysed together. The table also presents *p*-values (*n* = 50). Acoustic rhinometry results V2–5 cm had one missing patient (*n* = 48)

**Fig. 3:**
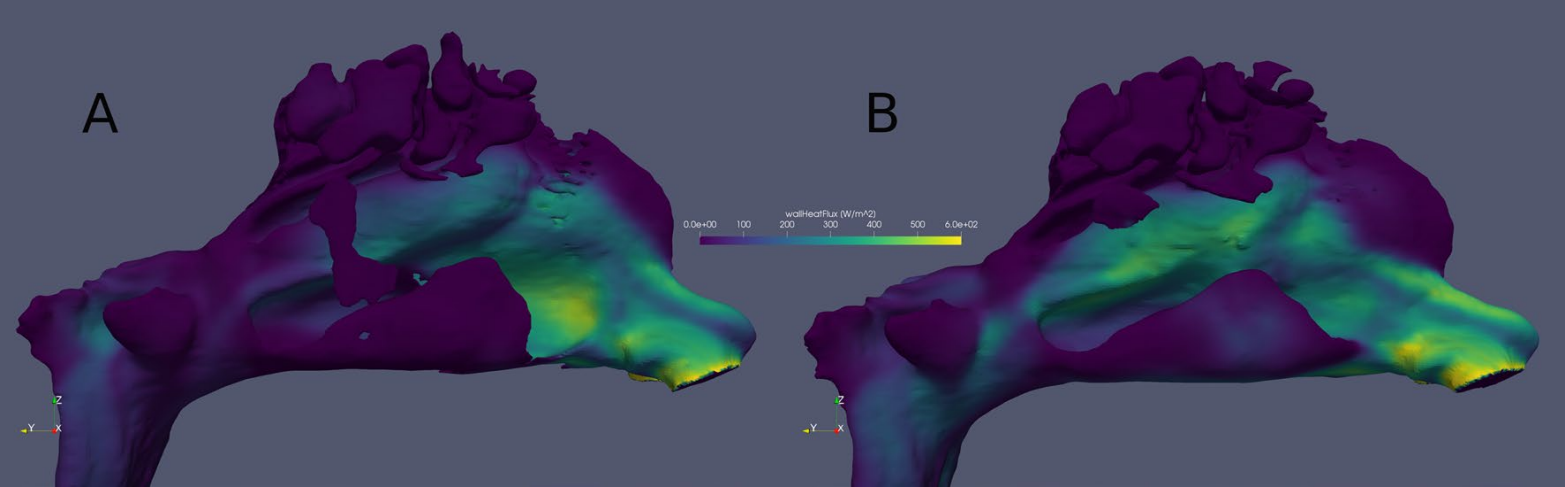
The pre- (**A**) and postoperative (**B**) nasal mucosa heat flux (W/m^2^) results for one patient

In the anterior parts of the inferior turbinates, a statistically significant negative correlation was found between total wall shear force value changes against volumetrically measured air volume changes (*n* = 25) between the pre- and postoperative conditions (*p* < 0.001). In the same area, heat transfer changes against air volume changes were not statistically significant. Furthermore, no statistically significant correlations were found between total wall shear force or heat transfer changes against acoustic rhinometry V2–5 cm changes. For patients’ subjective assessment, there was a statistically significant positive correlation in the anterior parts of the inferior turbinates between total wall shear force changes against nasal obstruction VAS changes (*p* = 0.04). No other statistically significant results were found between total wall shear force or heat transfer changes against nasal obstruction VAS or GHSI changes. (Table 3) The pre- and postoperative mucosal heat fluxes are visually presented for one patient **(**Fig. 3**)**.

**Table 3:** Univariate Spearman correlations between changes in calculated CFD values (total wall shear forces and total heat transfer) from the anterior 5–20 mm parts of the inferior turbinates and changes in subjective and volumetric measures

Spearman correlations between pre- and postoperative changes in CFD and changes in other subjective and objective measures in the anterior 5–20 mm parts of the inferior turbinates								
	Wall shear forces against VAS	Heat transfer against VAS	Wall shear forces against GHSI	Heat transfer against GHSI	Wall shear forces against V2–5 cm	Heat transfer against V2–5 cm	Wall shear forces against air volume	Heat transfer against air volume
Spearman’s rho	0.40	0.25	− 0.19	0.06	− 0.24	0.12	− 0.65	− 0.36
*p*-value	0.04	0.23	0.38	0.77	0.27	0.58	< 0.001	0.08

Changes in CFD values are calculated by subtracting preoperative values from 12-month postoperative values. Both sides of the nasal cavities are analysed together. The table also presents *p*-values (*n* = 25). Acoustic rhinometry results V2–5 cm had one missing patient (*n* = 24)

**Fig. 4:**
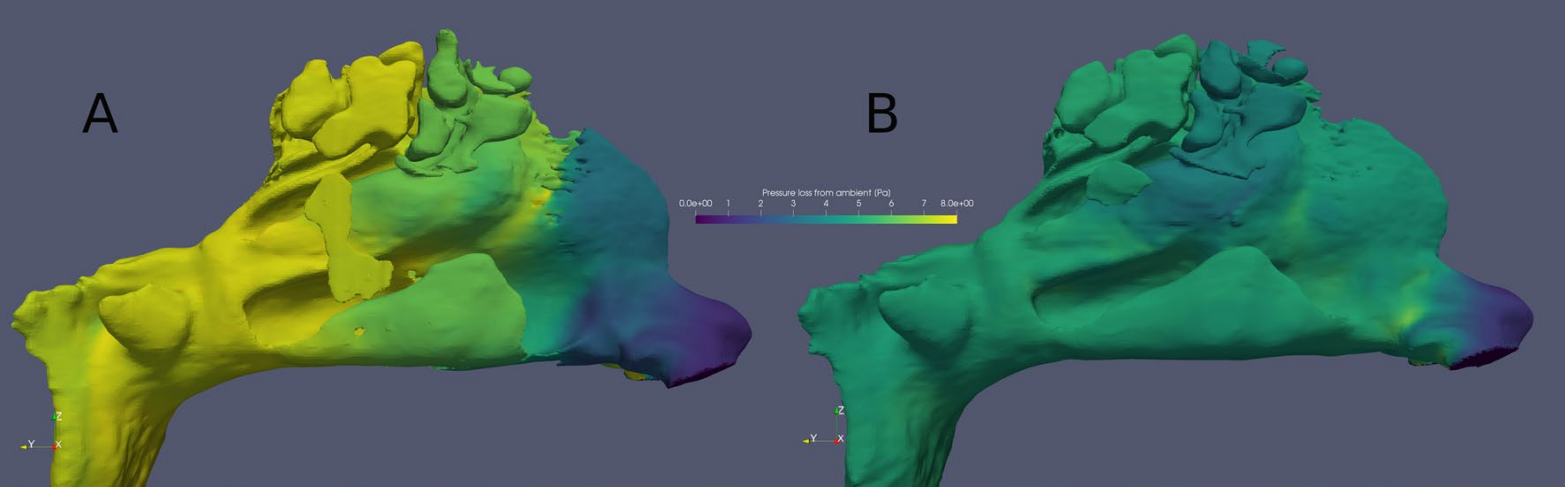
The pre- (**A**) and postoperative (**B**) pressure loss (Pa) results from ambient to the nasal mucosa for one patient

Further, no statistically significant correlations were found between pressure losses from ambient to nasopharynx (*n* = 50) and their changes (*n* = 25) between the pre- and postoperative conditions against subjective nasal obstruction VAS and GHSI measures. However, a statistically significant positive correlation was found between pressure loss changes (*n* = 25) along the nasal cavity against total wall shear force value changes in the operated parts of the inferior turbinates between the pre- and postoperative conditions. (Table 4) The pre- and postoperative pressure results are visually presented for one patient **(**Fig. 4**)**.

**Table 4:** Univariate Spearman correlations between calculated CFD pressure loss from ambient to nasopharynx against subjective measures and total wall shear stresses in the anterior 5–20 mm parts of the inferior turbinates

Spearman correlations between CFD calculated pressure losses against subjective measures and wall shear forces in the anterior 5–20 mm parts of the inferior turbinates					
	Pressure loss along the nasal cavity against VAS (*n* = 50)	Pressure loss along the nasal cavity against GHSI (*n* = 50)	Pressure loss changes along the nasal cavity against VAS changes (*n* = 25)	Pressure loss changes along the nasal cavity against GHSI changes (*n* = 25)	Pressure loss changes along the nasal cavity against changes in wall shear forces in the anterior 5–20 mm parts of the inferior turbinates (*n* = 25)
Spearman’s rho	0.02	0.04	0.16	0.04	0.64
*p*-value	0.86	0.79	0.45	0.86	< 0.001

Changes in CFD values are calculated by subtracting preoperative values from 12-month postoperative values. Both sides of the nasal cavities are analysed together. The table also presents *p*-values (*n* = 50, *n* = 25)

## Discussion

In this study, we have presented the CFD results and air volumes and corresponding acoustic rhinometry results from inferior turbinate surgeries both preoperatively and at 12 months postoperatively for what was to our knowledge the largest cohort of patients to date. The aim of inferior turbinate surgery is to reduce turbinate volumes and increase airway volumes in the operated parts. Our CFD results indicate statistically significant decreases in total wall shear forces in the anterior parts of the inferior turbinates postoperatively. Furthermore, statistically significant negative correlations were found between total wall shear forces and volumetrically measured absolute air volumes in the operated parts as well as in their changes between the pre- and postoperative conditions.

In the previous literature, Kimbell et al. [5] evaluated patients who had undergone septoplasty and found that average WSS increased in the obstructed side of the nose postoperatively. However, that study used a different study setting to the one used in our study. For example, in their study, average WSS was calculated from the whole obstructed cavity and the patient-specific pressure drop across the nasal cavities was assumed to be equal between the pre- and postoperative conditions. In their results, the less obstructed side also had higher average WSS postoperatively, albeit not statistically significantly. Their results were affected by smaller postoperative mucous membrane surface areas. Therefore, average WSS is likely expected to increase with the assumption that pressure drops across the nasal cavities do not change between the pre- and postoperative conditions. Without this assumption, our results reveal that bilateral inferior turbinate surgery leads to larger air volumes and reduced total wall shear forces in the operated anterior parts postoperatively.

For patients’ subjective assessment, our results reveal there were statistically significant total wall shear force changes in the anterior parts of the inferior turbinates against VAS changes (*n* = 25) between the pre- and postoperative conditions. Based on our results, it is unclear whether smaller wall shear force values contribute to better VAS subjective improvement because of smaller pressure drops along the nasal cavity or because of mechanical subjective sensing of reduced wall shear forces at the surface mucous membrane in the anterior parts of the nasal cavities. As wall shear forces act as a mechanical force on the surface of the mucous membrane, it also affects the mechanical sensing of the nasal airflow. Our nasal obstruction VAS changes against pressure drop results from ambient to nasopharynx did not obtain as significant results as the decreased wall shear forces in the anterior parts of the inferior turbinates.

Several virtual studies of turbinate surgery have, in most cases, shown decreased total heat transfer [17–19]. In virtual studies, artificial bilateral theoretical surgery is performed, and the effects of the surgery are evaluated computationally. Similarly, for real life patients, our pilot study [14] reported reduced heat transfer results in the operated parts for inferior turbinate surgery postoperatively. In the present study, the heat transfer in the operated parts did not decrease significantly. With a greater number of patients, however, our results could have possibly shown statistical significance in those results. In this study, similar negative correlations were found in the anterior parts of the inferior turbinates between heat transfer against absolute air volumes and their changes between the pre- and postoperative conditions. However, no statistical significance was found.

Previously, the results of other pre- and postoperative CFD studies have been reported after concomitant septorhinoplasty or septoplasty and turbinate surgery in patients with turbinate hypertrophy and septum deviation. In those studies, the CFD results were usually analysed for the whole nasal cavities separately for the most obstructed side and for the more unobstructed side. These studies have shown increased heat fluxes or total heat transfer from the most obstructed side of the nose postoperatively [5–8]. Sullivan et al. [6] conducted a study pre- and postoperatively without healthy controls. They reported increased total heat transfer and heat fluxes postoperatively in the most obstructed side. In addition, they also reported a surface area, where heat flux exceeds 50 W/m^2^ and peak heat flux. The best heat transfer correlations were obtained with surface areas against subjective measures. Other studies have reported heat fluxes but not the heat transfer rates reported in the present study. Kimbell et al. [5] reported pre- and postoperative heat fluxes for patients (*n* = 10) from a larger cohort who did not have nasal cycle effects in CT images. In contrast, Gaberino et al. [7] studied patients with nasal cycle effects pre- and postoperatively. Heat fluxes and surface area, where heat flux exceeds 50 W/m^2^, were reported. Nasal cycle effects were corrected computationally and correlations between CFD variables and subjective scores improved. Na et al. [8] studied bilateral septoturbinoplasty (*n* = 8) and found a correlation between the NOSE scores and with an increase in surface heat flux from the most obstructed side.

In all the above-mentioned studies, it is natural that septoplasty operations led to a more uniform airflow distribution between the nasal cavities. Therefore, heat fluxes increased postoperatively in the most obstructed side. Furthermore, volume flow rates across the nasal cavities increased postoperatively, as it is assumed that postoperative pressure loss was equal to the preoperative pressure loss, resulting in reduced flow rates preoperatively [5–8]. However, with this assumption, the surgical procedure itself affects the calculated preoperative volume flow rates and preoperative CFD results. This effect can also be partly observed in heat transfer results, which are different from those in the present study. Furthermore, in other studies, analysis is evaluated on a greater surface area compared to the anterior parts of the inferior turbinates where our surgical operations were performed.

Our study indicates reduced heat transfer in the operated parts postoperatively but without statistical significance. In our results, it was assumed that inspiratory flow rates are independent from the volumetric changes caused by surgery and that patients breathe as much pre- and postoperatively as corresponding healthy individuals as a function of gender and weight. It can be expected, therefore, that this assumption holds true for a large majority of patients without excessive nasal deformities.

Casey et al. [9] examined preoperative patients and healthy controls but did not evaluate the effects of the surgical procedure. In their study, a constant volume flow rate of 15 l/min was assumed for both patients and healthy controls. Preoperative patients and healthy controls were defined from VAS scores. The study found that preoperative patients had smaller average heat flux in the most obstructed side than healthy controls. Furthermore, they also found that healthy subjects had a significantly higher middle airflow than preoperative patients on the most obstructed side. In our study, the results in the middle parts of the nasal cavities were not analysed separately. It is probable that septum deviations affected their results compared to our cohort. Since inferior turbinate surgery increases airway volumes and airflow in the inferior parts, middle airflow and corresponding heat transfer could be expected to decrease without septum deviations. Our results were concentrated on the inferior turbinate region where the surgical operations were performed. In future, middle airflow regions could be investigated in more detail in studies with larger cohorts.

In most other studies, NOSE scores obtained greater or similar CFD correlations than nasal obstruction VAS scores against CFD variables [5–7, 9]. In our study, general QOL results were collected from the GHSI questionnaire instead of from NOSE surveys. It remains unclear, however, why our GHSI questionnaire did not have comparable correlations as VAS results against objective CFD measures. It might be that the patient’s septoplasty operations contributed to the patients’ QOL more than the similar bilateral surgical operations performed in our patient cohort. Casey et al. [9] reported that a stronger correlation was found between NOSE survey results against VAS results from the most obstructed side than from the more unobstructed side. Our patient cohort was different and the surgical operations were bilateral.

In previous studies [5–7, 9], instantaneous patient VAS results were investigated unilaterally while the other side of the nose was blocked. This is, however, different from CFD calculations, where the nasal airflow is bilateral. It is challenging to evaluate how well patients’ subjective assessment changes from natural breathing conditions to an instantaneous unilateral subjective assessment of the severity of nasal obstruction. In our study, we did not use instantaneous nasal obstruction VAS assessment separately for both sides of the nasal cavities. Instead, the patients’ simultaneous nasal congestion VAS assessment from both sides of the nasal cavities was collected from the previous 7 days. The choice of different VAS assessment is challenging and depends greatly on the patient cohort studied.

To the best of our knowledge, our study had the most clinically homogenous patient cohort in CFD studies to date. Furthermore, our surgical procedure and patients’ subjective assessment of the severity of nasal obstruction differ in several important ways from previous study settings. Our surgical procedure was concentrated on the anterior parts of the inferior turbinates. Therefore, the operated parts were smaller and the surgical procedures performed were more similar than septoplasty patients have previously undergone. In our results, the correlations between VAS changes and heat transfer changes in the operated parts were not statistically significant. However, wall shear force value changes in the operated parts had significant correlations against patients’ subjective assessment. Based on the findings of our study, wall shear force changes could be at least as informative as heat transfer in the patients’ subjective assessment of the severity of nasal obstruction. Future studies should be concentrated on studying and reporting these results in more detail.

## Conclusion

Inferior turbinate surgeries in the operated anterior parts of the inferior turbinates lead to decreased total wall shear force values postoperatively. Changes in subjective nasal obstruction VAS results against total wall shear force changes in the operated parts between the pre- and postoperative conditions were statistically significant. CT imaging information could be useful in future for performing CFD calculations in clinical practice. CFD data have a potential to be used for evaluation of nasal airflow.

## Data Availability

All data are available on request.
